# Combined use of expression and CGH arrays pinpoints novel candidate genes in Ewing sarcoma family of tumors

**DOI:** 10.1186/1471-2407-9-17

**Published:** 2009-01-14

**Authors:** Suvi Savola, Arto Klami, Abhishek Tripathi, Tarja Niini, Massimo Serra, Piero Picci, Samuel Kaski, Diana Zambelli, Katia Scotlandi, Sakari Knuutila

**Affiliations:** 1 Department of Pathology, Haartman Institute and HUSLAB, University of Helsinki, Helsinki, Finland; 2 Helsinki University Central Hospital, Helsinki, Finland; 3 Department of Information and Computer Science, Helsinki University of Technology, Espoo, Finland; 4 Laboratorio di Ricerca Oncologica, Istituti Ortopedici Rizzoli, Bologna, Italy

## Abstract

**Background:**

Ewing sarcoma family of tumors (ESFT), characterized by t(11;22)(q24;q12), is one of the most common tumors of bone in children and young adults. In addition to *EWS/FLI1 *gene fusion, copy number changes are known to be significant for the underlying neoplastic development of ESFT and for patient outcome. Our genome-wide high-resolution analysis aspired to pinpoint genomic regions of highest interest and possible target genes in these areas.

**Methods:**

Array comparative genomic hybridization (CGH) and expression arrays were used to screen for copy number alterations and expression changes in ESFT patient samples. A total of 31 ESFT samples were analyzed by aCGH and in 16 patients DNA and RNA level data, created by expression arrays, was integrated. Time of the follow-up of these patients was 5–192 months. Clinical outcome was statistically evaluated by Kaplan-Meier/Logrank methods and RT-PCR was applied on 42 patient samples to study the gene of the highest interest.

**Results:**

Copy number changes were detected in 87% of the cases. The most recurrent copy number changes were gains at 1q, 2, 8, and 12, and losses at 9p and 16q. Cumulative event free survival (ESFT) and overall survival (OS) were significantly better (P < 0.05) for primary tumors with three or less copy number changes than for tumors with higher number of copy number aberrations. In three samples copy number imbalances were detected in chromosomes 11 and 22 affecting the *FLI1 *and *EWSR1 *loci, suggesting that an unbalanced t(11;22) and subsequent duplication of the derivative chromosome harboring fusion gene is a common event in ESFT. Further, amplifications on chromosomes 20 and 22 seen in one patient sample suggest a novel translocation type between *EWSR1 *and an unidentified fusion partner at 20q. In total 20 novel ESFT associated putative oncogenes and tumor suppressor genes were found in the integration analysis of array CGH and expression data. Quantitative RT-PCR to study the expression levels of the most interesting gene, *HDGF*, confirmed that its expression was higher than in control samples. However, no association between *HDGF *expression and patient survival was observed.

**Conclusion:**

We conclude that array CGH and integration analysis proved to be effective methods to identify chromosome regions and novel target genes involved in the tumorigenesis of ESFT.

## Background

The Ewing sarcoma family of tumors (ESFT) is a group of highly aggressive and often metastatic small round cell tumors characterized by specific t(11;22)(q24;q12) chromosomal rearrangements, which create the *EWS/FLI1 *gene fusion and thereby a chimeric, oncogenic transcription factor [1]. ESFT is one of the most common bone and soft tissue tumors in children and young adults arising generally during the second decade of life [2,3]. The ESFT tumors are divided into four subtypes according to the histopathological description: classical Ewing sarcoma in bones, extraskeletal Ewing sarcoma, peripheral neuroepithelioma (PNET), and Askin's tumor. Most of these ESFT cases manifest defects in the maintenance of genomic stability with subsequent DNA copy number alterations.

Conventional CGH and array CGH studies have shown that 63–84% of ESFT patient samples have copy number changes [4-9]. These copy number alterations play a significant role in the tumorigenesis and malignant progression of solid tumors. The diagnosis and clinical management of patients would substantially benefit from identification of these novel chromosomal targets and molecular markers involved in the tumorigenesis of ESFT, since secondary genetic alterations in ESFT have been shown to correlate with patient's outcome. In addition to overall number of chromosomal imbalances [10,11], gains of 1q, 8 and 12 and losses of 9p21.3 and 16q have been associated with poor clinical outcome [7,12-14]. Rapid development of microarray technology has led to more sophisticated analyses, which can be utilized to find novel tumor specific genetic alterations. Further, numerous studies have demonstrated that integrating genomic data from different sources, e.g. at RNA and DNA level, can enhance the reliability of genetic analysis in understanding tumor progression. Our aim was to identify common regions of gain and loss and to define the influence of copy number alterations on gene expression to identify chromosomal areas and genes involved in malignant progression of Ewing sarcoma. We used high-resolution array-based CGH to screen simultaneously multiple loci for possible copy number imbalances in ESFT patient samples. This approach enables us to detect both large-scale and gene-size copy number alterations down to ~35 kb in size. To investigate the impact of copy number imbalances on the gene expression levels of affected genes, we performed also an expression array analysis to combine RNA and DNA level data and validated the most interesting result by quantitative RT-PCR analysis.

## Methods

### Patient samples and clinical data

Fresh frozen samples (stored at -70°C) were collected from the archives of the Laboratory of Oncologic Research, Istituti Ortopedici Rizzoli (IOR), Bologna. A total of 31 tumor specimens of from ESFT patients treated at IOR between years 1992 and 2005 were available for the aCGH study. In order to study ESFT expression profiles, 42 patient samples were collected for RNA extraction. To validate the ESFT diagnosis, the presence of *EWS/FLI *or *EWS/ERG *translocation was confirmed by RT-PCR for all samples with available RNA. Clinical data for 31 samples (Table 1) used in aCGH and in data integration analysis were collected from the patient records at IOR. All patients were treated within controlled prospective trials [15,16]. The mean age of the patients was 20.7 years, ranging from 5 to 41 years and the male-to-female ratio was 22:9 (2.4). Of the 31 samples used in aCGH analysis, 23 were primary tumors, two recurrencies, and six metastatic tumors. The majority of these patients (22/31) were diagnosed with classical Ewing sarcoma, four with soft tissue Ewing sarcoma, three with Askin's tumor and two with PNET. Seven of the patients with primary tumors had metastases at the time of diagnosis. Sixteen tumors had Type 1 (exon 7 of *EWS*/exon 6 of *FLI1*) gene fusion, eight had other types of fusion (Type 2: exon 7 of *EWS*/exon 5 of *FLI1 *or Type 3: exon 10 of *EWS*/exon 6 of *FLI1*), three samples were negative for the most common fusion genes (*EWS-FLI1 *and *EWS-ERG*), and in four cases this information was not available. The sample set was handled in a coded fashion and the collected clinical and quality control data of the samples is publicly available in a microarray database at http://www.cangem.org[17]. This study has been reviewed and approved by the Ethical Review Board of Helsinki University Central Hospital.

**Table 1:** Clinical data of the ESFT patients included in array CGH and/or data integration analysis.

Code	Included in data integration analysis	Diagnosis	Origin of sample	Chemotherapy or radiation before sample collection	Material from	Necrosis	Patient Status at Diagnosis	Age	Sex	Location	Outcome EFS	EFS months	Outcome OVS	OVS months	Type of Translocation
D150	X	Ewing	PRI	no	Biopsy		Localized	23	M	Femur	REL	6	DEAD	12	EWS/FLI-1 type 2
D152	X	Ewing	PRI	no	Biopsy		Pri Met Lung	15	M	Pelvis	REL	8	DEAD	17	EWS/FLI-1 type 2
D153	X	Ewing	PRI	no	Biopsy		Pri Met Bone	22	M	Pelvis	REL	0	DEAD	5	EWS/FLI-1 type 3
D154	X	Ewing	PRI	yes	Resection	50%	Localized	19	M	Humerus	REL	19	AWD	57	Neg/Neg
D155		Ewing	PRI	no	Biopsy		Localized	15	F	Pelvis	NED	69	NED	69	EWS/FLI-1 type 1
D162		Ewing	PRI	yes	Resection	90%	Localized	20	M	Tibia	NED	61	NED	61	Neg/Neg
D239		Ewing	PRI	yes	Resection	95%	Localized	21	F	Scapula	REL	34	NED	41	EWS/FLI-1 type 2
D240		Ewing	PRI	yes	Resection	30%	Pri Met Lung	10	F	Humerus	REL	13	DEAD	15	EWS/FLI-1 type 1
D241		Ewing	PRI	yes	Resection	> 95%	Localized	28	M	Femur	NED	47	NED	47	EWS/FLI-1 type 2
D245		Ewing	PRI	yes	Resection	70%	Localized	22	M	Radius	REL	4	DEAD	8	NA
D248		Ewing	PRI	yes	Resection	95%	Localized	11	M	Scapula	NED	76	NED	76	EWS/FLI-1 type 1
D250		Ewing	PRI	yes	Resection	75%	Pri Met Lung	32	F	Clavicula	REL	20	DEAD	31	Neg/Neg
D311	X	Ewing	PRI	no	Biopsy		Localized	13	M	Pelvis	NED	43	NED	43	EWS/FLI-1 type 1
D313	X	Ewing	PRI	no	Biopsy		Localized	24	M	Femur	REL	18	NED	117	EWS/FLI-1 type 1
D316	X	Ewing	PRI	yes	Resection	60%	Pri Met Lung	11	M	Femur	REL	10	DEAD	10	EWS/FLI-1 type 1
D320	X	Ewing	PRI	no	Biopsy		Localized	5	M	Humerus	REL	14	DEAD	21	EWS/FLI-1 type 2
D322	X	Ewing	PRI	yes	Resection	25%	Localized	27	F	Femur	REL	29	NED	67	EWS/FLI-1 type 1
D242		Ewing Extr	PRI	no	Biopsy		Localized	41	F	Thorax	NED	56	NED	56	NA
D246		Ewing Extr	PRI	no	Biopsy		Localized	26	F	Thigh	NED	49	NED	49	EWS/FLI-1 type 1
D249		Ewing Extr	PRI	no	Biopsy		Localized	35	M	Thigh	REL	7	AWD	13	NA
D252		Ewing Extr	PRI	no	Biopsy		Pri Met Lung	18	M	Thigh	REL	0	DEAD	13	EWS/FLI-1 type 1
D253		Askin's	PRI	yes	Resection	10%	Localized	24	M	Rib	REL	5	DEAD	12	EWS/FLI-1 type 2
D254		Askin's	PRI	yes	Resection	15%	Pri Met Lung	21	M	Rib	NA	NA	NA	NA	NA
D156		Askin's	REC	no	Resection		Localized	15	M	Rib	REL	17	DEAD	21	EWS/FLI-1 type 1
D157	X	Ewing	REC	no	Amputation		Pri Met Lung	30	F	Femur	REL	3	DEAD	25	EWS/FLI-1 type 1
D255	X	Ewing	LUNG MET	no	Resection		Pri Met Lung	17	M	Scapula	REL	31	DEAD	43	EWS/FLI-1 type 1
D256	X	Ewing	LUNG MET	no	Resection		Localized	34	M	Humerus	REL	17	NED	136	EWS/FLI-1 type 1
D312	X	Ewing	LUNG MET	no	Resection		Localized	9	M	Fibula	REL	28	DEAD	42	EWS/FLI-1 type 1
D321	X	Ewing	LUNG MET	no	Lobectomy		Localized	24	M	Pelvis	REL	34	DEAD	80	EWS/FLI-1 type 1
D315	X	PNET	BONE MET	no	Biopsy		Localized	16	M	Tibia	REL	58	DEAD	99	EWS/FLI-1 type 1
D257	X	PNET	BONE MET	no	Biopsy		Localized	16	F	Femur	REL	6	DEAD	8	EWS/FLI-1 type 2

Extr = extraskeletal; PRI = primary tumor; REC = local recurrence; MET L = metastasis in lung; MET B = metastasis in bone; EFS = event-free survival; REL = relapsed; NED = no evidence of disease; NA = not available; OVS = overall survival; AWD = alive with disease.

### Nucleic acid isolation

Genomic DNA from 31 samples was extracted using the standard phenol-chloroform method. Prior to extraction, the proportion of tumor cells was verified to exceed 75% in all samples by using hematoxylin and eosin-stained sections. Tissue necrosis was evaluated on the whole tumor mass. In cases with high percentages of necrosis, nucleic acids were isolated from the tissue samples in which viable cells were still present. Reference DNAs, male and female, were extracted from pooled blood samples (4 individuals) obtained from Blood Service, Red Cross, Finland. RNA from 42 ESFT samples was isolated using a TRIzol extraction kit (Invitrogen Ltd., Paisley, UK) according to the manufacturer's instructions. Both high quality genomic DNA and RNA were available for 16 patients after the nucleic acid extraction instead of 42 patients, due to insufficient amount of starting material. DNA and RNA concentrations were measured using a GeneQuant pro spectrophotometer (Amersham Pharmacia, Cambridge, UK), and RNA quality was assessed using Agilent's 2100 Bioanalyzer (Agilent, Palo Alto, CA).

### Array CGH hybridization, microarray image and data analysis

Digestion, labeling, hybridization, and data analysis of genomic DNA was performed according to Agilent's protocol version 2.0 for 44K arrays as described previously [18,19]. In brief, the sample and reference DNAs, 7 μg each, were fragmented and 1.0–1.5 μg of the fragmented DNA was labeled by random priming using a BioPrime array labeling kit (Invitrogen, Carlsbad, CA) with Cy3-dUTP and Cy5-dUTP dyes (Perkin-Elmer, Wellesley, MA). Labeled samples were purified, combined, and hybridized for 48 h at 65°C, 10 rpm to Human Genome CGH 44B oligomicroarray slides (Agilent Technologies Santa Clara, CA) against gender matched reference DNAs. Then the arrays were washed and scanned [18]. The array images were analyzed and data was extracted using Agilent's Feature Extraction (FE) Software version 8.1, providing dye normalization (Linear Lowess) and background substraction. The chromosomal imbalances were identified using Agilent's CGH Analytics software version 3.4. The altered chromosomal regions and breakpoints were detected using ADM-2 (threshold 8.0) with 1.0 Mb window size. Patient survival analysis was then performed by Kaplan-Meier and Logrank (Mantel-Cox) methods considering either event-free or overall survival.

### Expression array hybridizations

The ESFT RNA samples, 42 cases and control samples, a CD34+ cell line and a pool of normal muscle tissue samples were hybridized to Affymetrix Human Genome U133 Plus 2.0 oligonucleotide microarrays (Affymetrix, Santa Clara, CA) according to the manufacturer's GeneChip^® ^One-Cycle Target Labeling-protocol. In brief, 5 μg of total RNA was reverse transcribed to cDNA using One-Cycle cDNA Synthesis Kit (Affymetrix). Biotin-labeling of antisense cRNA was carried out using IVT Labeling Kit (Affymetrix). The labeled and fragmented cRNA (15 μg of each) was hybridized for 16 h at 45°C in a hybridization oven 640 (60 rpm). Washing and staining of the arrays with streptavidin-phycoerythin (SAPE) was completed in a Fluidics Station 450 (Affymetrix). The arrays were then scanned using a confocal laser GeneChip Scanner 3000 and images were analyzed using GeneChip Operating Software (GCOS; Affymetrix, Sacramento, CA). The expression measurements were preprocessed using Robust Multi-array Analysis (RMA) for the whole collection of 44 chips (42 ESFT patients and two hypothetical normal samples). While only 16 of these were used in the integration, running the preprocessing for the whole collection (n = 44) provides more accurate estimates of the true expression levels.

### Integration of gene copy number and expression data

In order to compare the measurements obtained on Affymetrix and Agilent platforms, the sequences used in the probes were matched to the NCBI36 human genome build, using the BLAST algorithm to provide a unique location for each Affymetrix probe set using the target sequences provided by Affymetrix and each Agilent probe. Multiple matches were combined to provide a single location covering all matches if the resulting sequence length was below 2,5 Mb. Note that the locations do not necessarily match the reference sequences of the NCBI36 genome, since they correspond to the locations of the probe sequences, not RefSeqs. In the joint analysis, each Affymetrix probe set was paired with the closest Agilent probe, measured as the distance between the mean points of the sequences. The Affymetrix probe sets that had no Agilent probes within 375 kb were ignored. Correlation between expression and gene copy number of different patients was measured separately for each gene (identified based on the Affymetrix probe set). Genes with high positive or negative correlation were chosen for further examination. The goal of this process was to detect genes where a copy number change and a change in expression are observed on the same patients. A similar analysis was conducted by first dividing patients into groups according to their copy number status and then testing whether these groups have a significant difference in their expression levels [20]. Here the correlation approach was chosen instead of the testing approach, because it can take into account also small amplification imbalances not detectable with the method described in Section "Array CGH hybridization, microarray image and data analysis". It also takes into account possible higher copy number changes. As a correlation measure we used Spearman's rank correlation, since the copy number data does not follow normal distribution. We used the algorithm of Best [21] to compute the p-value against the correlation being zero, and corrected for multiple testing by computing false discovery rates using the q-value procedure [22]. The correlation was computed only for genes located on chromosome arms where at least 20% of the full patient collection, including also samples not used in the integration analysis, showed copy number aberration, in order to focus on regions where associations would be likely.

### Quantitative RT-PCT analysis by TaqMan Low Density Arrays

Pre-designed TaqMan PCR probe and primer sets for *HDGF *were used: Assay ID Hs00610314-m1 (Applied Biosystems, Foster City, CA, USA). All PCRs were done by using ABI PRISM 7900 Sequence Detection System (Applied Biosystems) as recommended by the supplier. Thermal cycling conditions were: 50°C for 2 min, 95°C for 10 min, 95°C for 15 sec and 60°C for 1 min. Gene expression values were calculated based on the ΔΔCt method [23], in which RNA from CD34+ cells derived from human bone marrow and pooled muscle normal tissues derived from three patients were the designated calibrators for the analysis of all other samples. CD34+ positive cells and pooled muscle normal tissues were processed in the same way as tumor samples and used as separate calibrators for the RT-PCR experiments. For evaluating the prognostic value of *HDGF*, we calculated its median expression value, and patients were stratified as "high-expressors" or "low-expressors" relative to the median value. Patient survival analysis was then performed by Kaplan-Meier and Logrank methods considering either event-free survival or overall survival.

## Results

### Copy number changes

**Figure 1:**
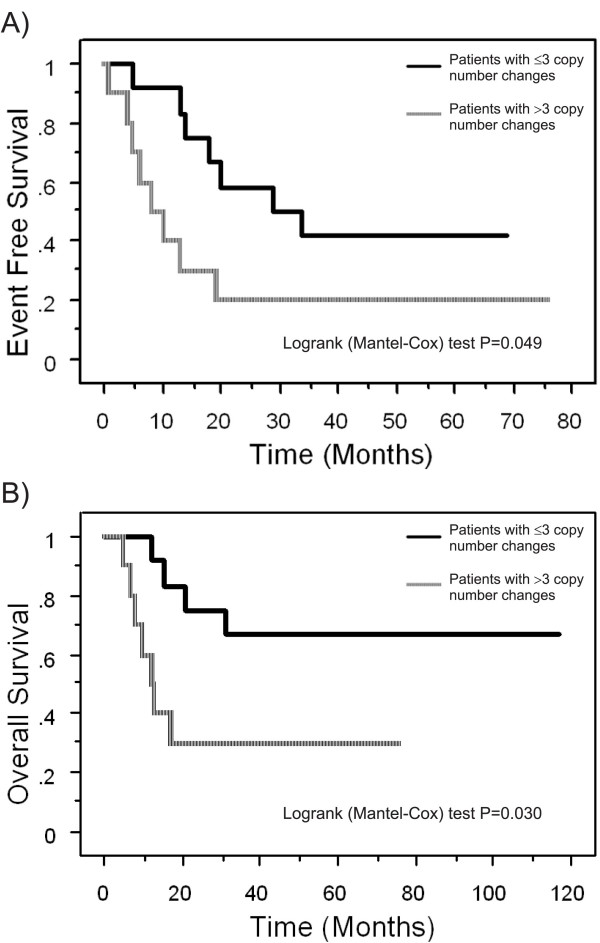
**Outcome of patients with low copy number changes (≤ 3 copy number aberrations) and high copy number changes (> 3 copy number aberrations)**. Kaplan-Meier plots show A) event-free survival and B) overall survival of patients with low copy number changes (≤ 3 copy number aberrations detected in the sample by array CGH) in bold line and with high number copy number changes (> 3 copy number aberrations) in hatched line.

**Figure 2:**
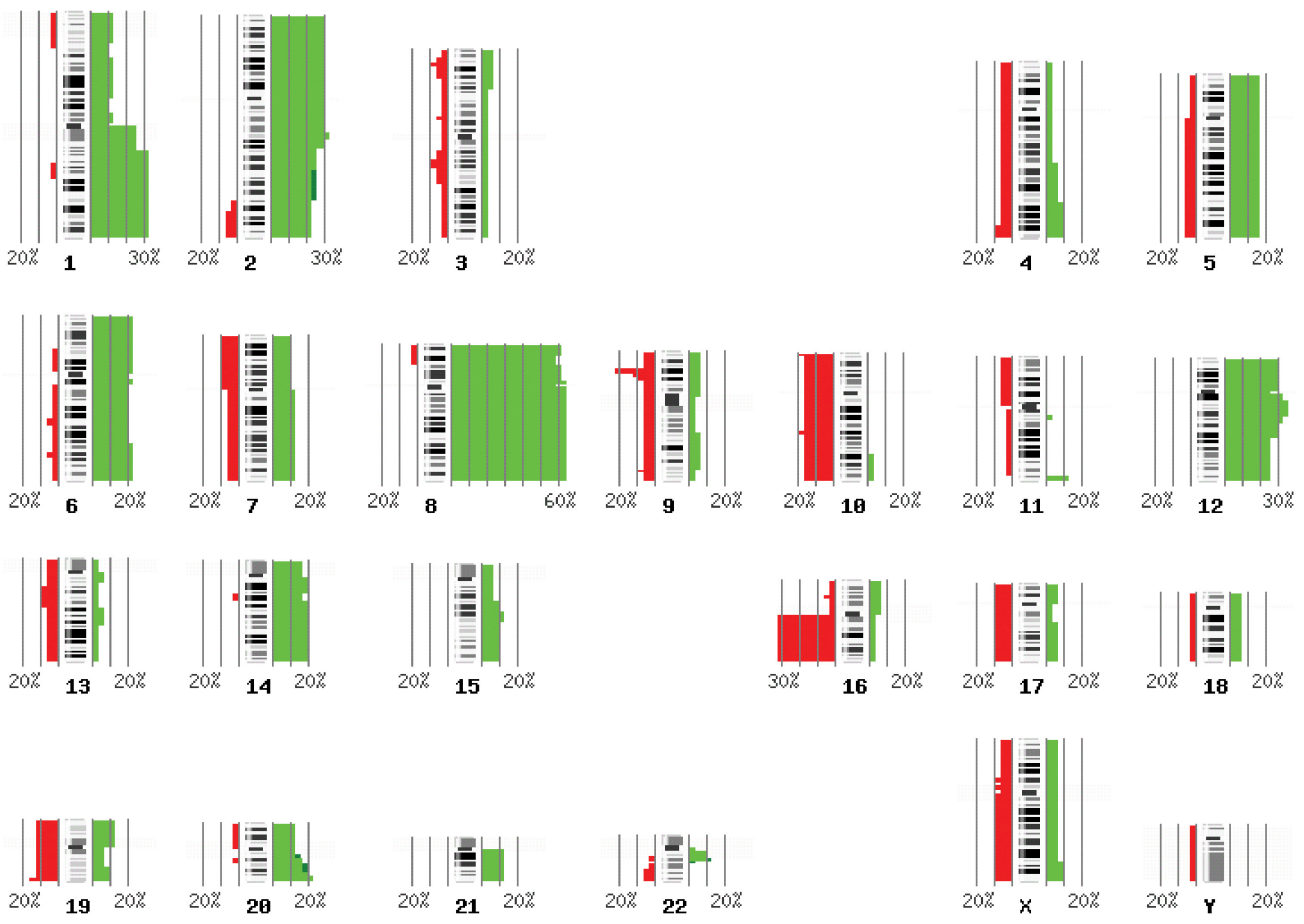
**Chromosomal locations of copy number changes in ESFT patient sample (n = 31)**. The ideogram shows the summary of gains and losses of DNA sequence copy numbers and their frequencies in ESFT tissue samples (n = 31) analyzed by array CGH. Gains (light green) and amplifications (dark green) are shown on the right of each chromosome and losses (red) on the left (number refer to the percentage per band). Chromosomal ideogram was generated using the PROGENETIX software [46].

**Figure 3:**
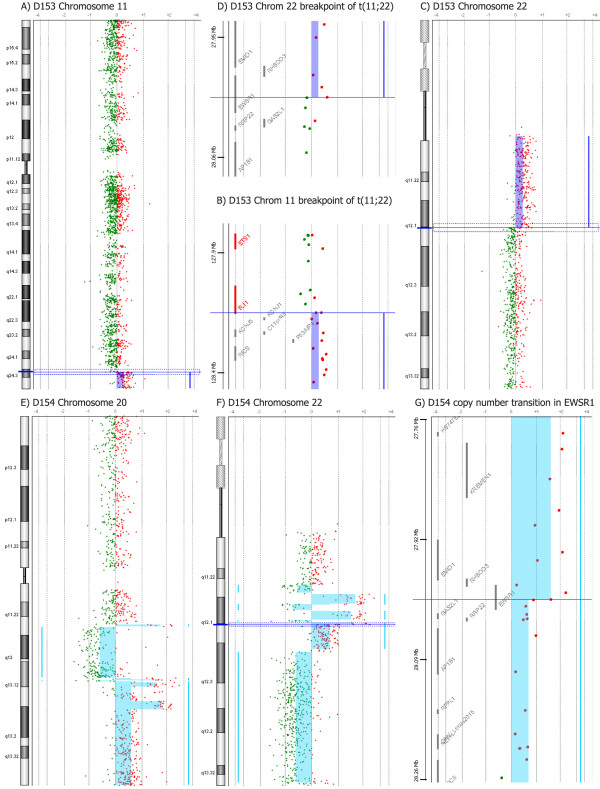
**Gain of chromosomal material on chromosomes 11 and 22 in patient sample D153 (A-D) and cryptic amplifications on chromosomes 20 and 22 in patient sample D154 (E-G)**. A) Chromosome 11 shows a gain of 11q24.3-qter. B) Breakpoint of copy number gain is inside the *FLI1 *locus. Based on aCGH results, the genomic breakpoint location is between 128148010 – 128186180. C) Chromosome 22 shows a gain of 22q11.21-q12.1. D) Breakpoint of copy number gain is inside the *EWSR1 *locus. Genomic breakpoint location is between 28007405 – 28007664. E) Chromosome 20 shows cryptic amplifications in 20q11.23, 20q13.12, and 20q13.12-q13.13, gain of 20q13.2-qter, and loss of 20q11.23-q13.12. F) Chromosome 22 shows loss of 20q11.23, uncontinuous amplifications in 22q11.23-q12.1 separated by segments of loss, gain of 20q12.2-q12.3, and loss of 20q12.3-qter. G) Copy number transition between amplification and gain in 20q12.1 is in the *EWSR1 *locus.

Results from a high-resolution analysis of copy number aberrations in ESFT (n = 31), using Agilent's 44K oligoarray platform and CGH analytics software are shown in Table 2. In all ESFT patient samples, 0–26 aberrations were detected per sample (mean: 7.2) and 27 of the 31 samples showed (87%) copy number changes. All samples without copy number changes (n = 4) were primary tumors. Metastases (mean: 11.8) showed more copy number changes than local recurrencies (mean: 9.5) and primary tumors (mean: 5.8). The sizes of these aberrations ranged from < 60 kb deletions to gains or losses of whole chromosomes. Among primary tumors, the samples with low copy number changes (≤ 3 copy number aberrations) showed a significantly better prognosis with respect to those with a high number of chromosomal alterations (> 3 copy number aberrations), both in terms of event-free and overall survival (Figure 1). Indeed, only 3/11 patients (27%) with less than three copy number changes developed metastases within 6 years from diagnosis in contrast with 8/10 (80%) of those with a high number of chromosomal alterations (P = 0,03 Fisher's test), indicating how the number of chromosomal alterations may have a highly prognostic significance despite the low number of patients here considered. Recurrent aberrations were gains of 1q (32%), 2 (29%), 8 (67%), and 12 (29%) and losses at 9p (23%) and 16q (32%) as visualized in Figure 2. The prominent deletion in 9p21.3 harboring *CDKN2A *tumor suppressor gene and microdeletions of these region have been previously described and discussed in a separate report by Savola *et al*. [18]. The gain of chromosome 8 was the most prominent copy number change in our sample set (21 of 31 cases). Gain of 8q arm (minimal common overlapping area) was present in all samples with chromosome 8 aberration. The minimal common overlapping area of copy number gain in chromosome 1 was 1q22-qter. In chromosome 12 the smallest common region of gain was 12q13.2-q14.1, which harbors two known oncogenes, *ERBB3 *and *CDK4*. Losses of 16q were observed in three cases together with 1q gain, suggesting the occurrence of an unbalanced t(1;16). Interesting copy number gains of 11q24.3-qter and 22q11.12-q12.1 starting or ending, respectively, at *FLI1 *and *EWSR1 *loci, were detected in patient samples D153 (Fig. 3A–D), D248, and D254 (Table 2). Copy number imbalances affecting the same loci were detected also in samples D154 (uncontinuous amplification 22q12.1-q12.1) (Fig. 3F–G), D312 (+11q24.3-qter), and D315 (-22q12.1) (see Table 2). Original microarray data, scanned images and FE output text files, are available at the public repository CanGEM http://www.cangem.org[17].

**Table 2:** Array CGH results (n = 31) in ESFT patient samples arranged by diagnosis.

Code	Diagnosis	Origin of sample	Array CGH results	Number of changes	*CDKN2A* deletion status^1^
D150	Ewing	PRI	+1q, +2, +4, +6, +7, +8, -10, +12, -16q, +17, -19, +20	12	No deletion 2
D152	Ewing	PRI	+5, +7, +8, +21	4	No deletion 2
D153	Ewing	PRI	+1, -2q35-qter, +6, +8, +9p, -9q34.11, -10, +11q24.3-qter, +12, +16p, +19, +20q12-qter, +22q11.21-q12.1	13	No deletion 2
D154	Ewing	PRI	-3p13, -3p25.1-p25.3, -6q21, -6q24.1-q24.2, +8p11.21-p21.2, -8p21.3-pter, +8q, -9q, amp20q11.23, -20q11.23-q13.12, amp20q13.12-q13.2, +20q13.2-qter, -22q11.23, amp22q12.1-q12.1 (uncontinuous), +22q12.2-q12.3, -22q12.3-qter	16	No deletion 2
D155	Ewing	PRI	No changes detected	0	No deletion 2
D162	Ewing	PRI	-9p21.2-p21.3	1	Heterozygous deletion 2
D239	Ewing	PRI	No changes detected	0	No deletion 2
D240	Ewing	PRI	+1q22-qter, +12, +Xq26.3-qter	3	No deletion 2
D241	Ewing	PRI	No changes detected	0	No deletion 2
D245	Ewing	PRI	+4q25-qter, +5, +8, +13q11-q12.2	4	No deletion 2
D248	Ewing	PRI	+1, +2q21.2-q33.1, amp 2q31.2-q33.1, -2q33.2-qter, -4, +8, del 9p21.3, -10, +11q24.3-qter, +12, +14, -16p12.3, -16q, +17p, -17q, +22q11.21-q12.1	16	Homozygous deletion 2
D250	Ewing	PRI	-3q13.31-q22.3, del 9p21.3, -Xp11.3	3	Homozygous deletion 2
D311	Ewing	PRI	+1q22-qter, +8, -10p15.3, -16q, +17q21.31-qter, -19q13.43	6	No deletion 3
D313	Ewing	PRI	+2, +8	2	No deletion 3
D316	Ewing	PRI	+2, +8, +14q11.2-q13.2, -14q13.3-q21.1, +14q21.1-qter, -X	6	No deletion 3
D320	Ewing	PRI	+8	1	No deletion 3
D322	Ewing	PRI	+8, -16q, +19p	3	No deletion 3
D242	Ewing Extr	PRI	+1q	1	No deletion 2
D246	Ewing Extr	PRI	No changes detected	0	No deletion 2
D249	Ewing Extr	PRI	-1p34.3-pter, +3p22.1-pter, -6p11.2-p21.31, +8, -10q23.1, +10q25.1-qter, -13q13.3-q14.3, +13q21.1-q22.1, +15q21.3-q22.2, +19q13.31-qter, +20q13.33	11	No deletion 2
D252	Ewing Extr	PRI	+2, +5, +6, -7, +8, -9p21.3, +12, +14, +21, -X	10	Homozygous deletion 2
D253	Askin's	PRI	+8, -Y	2	No deletion 2
D254	Askin's	PRI	+2, +3, -4q34.3-qter, -5, +6, -7, -9, del 9p21.3, -10, +11q24.3-qter, +12, -13, +14, +16, +18, +19, +20, +21, +22q11.21-q12.1	19	Homozygous deletion 2
D157	Ewing	REC	+2p, +2q11.2-q22.3, -3q13.13-q13.33, +4q31.3-qter, +8, +12p, +12q12-q21.31, -16q, -17p, -19, +X	11	No deletion 2
D156	Askin's	REC	+1q, +8p11.21-p11.22, +8p23.1-pter, +8q, +11q12.3-q13.2, +12q13.2-q14.1, -16q, +19p	8	No deletion 2
D255	Ewing	MET	+1q, +2, +5, +6, +8, +12, +13, +15, -16, -17, +18, -19, +20, +21	14	No deletion 2
D256	Ewing	MET	-1q24.2-q25.3, +8	2	No deletion 3
D312	Ewing	MET	+1, +2p, +2q11.2-q21.3, -3, -4, -5q, +6p, +6q12, -6q13-qter, +7q, +8, +9, -10, -11p, -11q12.1-q24.2, +11q24.3-qter, +12, -13, +14, +15q15.2-qter, -16q, -17, -18, -19, +20, +X	26	No deletion 3
D321	Ewing	MET	+2, +5, +6, +8, +14, +15, -16q, -20p, +21, -Xp11.21-p11.22, -Xq	11	No deletion 3
D257	PNET	MET	+1p11.2-p13.2, +1p22.1-p32.3, +1p35.1-pter, +1q, -3p24.3-p25.1, +6q23.1-qter, +7, +8q, -9p, +9q22.1-q33.3, +12p, +12q12-q15	12	Heterozygous deletion 2
D315	PNET	MET	-7p, -9p21.1-p21.3, del 9p21.3, -16q, -22q12.1, -22q13.2-q13.32	6	Homozygous deletion 3

^1^*CDKN2A *deletion was regarded as homozygous if the copy number log_2 _ratio was < -2.0 and as heterozygous if the ratio was -1.5 to -2.0. ^2^*CDKN2A *deletion result published previously by Savola et al. [18] in an article, which included total of 26 ESFT patient samples. ^3^*CDKN2A *deletion status not published before.

Extr = extraskeletal; PRI = primary tumor; REC = local recurrence; MET = metastasis.

### Integration of gene copy number and expression data

Array CGH data and expression data were combined for a total of 16 patient samples (Table 1). Matching of expression microarray probes to the corresponding copy number microarray probes using a 375 kb genomic window yielded 53,145 probe pairs. 10,115 of those located in chromosomal areas where at least 20% of patients showed a copy number aberration (1q, 2q, 8q, 12, and 16q). Several putative ESFT-related genes were pinpointed, differentially expressed due to copy number alteration in these chromosomal locations of highest interest. These novel putative oncogenes and tumor suppressor genes based on our data analysis include 20 genes (by q-value < 0.20), which previously have not been associated with ESFT (Table 3). For a supplementary table with all integration analysis results see Additional file 1.

**Table 3:** Putative target genes for tumorigenesis and tumor progression in recurrent copy number changes of the 16 ESFT patients included in the integration analysis.

Symbol	Name	Cytoband	Location^1^	Correlation	p-value	Copy number status^2^	q-value	Functional information
*HEATR3*	HEAT repeat containing 3	16q12.1	48697286–48697733	0,876	2,2E-16	Deleted	6,05E-13	NA
*TRAFD1*	TRAF-type zinc finger domain containing 1	12q24.12	111075038–111075556	0,853	2,2E-16	Gained	6,05E-13	[47]
*C1orf131*	Chromosome 1 open reading frame 131	1q42.2	229426179–229426672	0,850	2,2E-16	Unspecified	6,05E-13	NA
*HDGF*	Hepatoma-derived growth factor	1q21-q23	154978607–154979115	0,844	1,5E-05	Gained	0,024182394	[34,36-38]
*DDX47*	DEAD (Asp-Glu-Ala-Asp) box polypeptide 47	12p13.1	12873623– 12874134	0,844	1,5E-05	Gained	0,024182394	[48]
*GIGYF2*	GRB10 interacting GYF protein 2	2q37.1	233431003–233431539	0,838	3,5E-05	Unspecified	0,047741159	[49-51]
*C2orf49*	Chromosome 2 open reading frame 49	2q12.2	105331075–105331416	0,832	5,9E-05	Unspecified	0,069391132	NA
*TMEM63A*	Transmembrane protein 63A	1q42.12	224110806–224111244	0,829	7,3E-05	Gained	0,07491325	NA
*WDR67*	WD repeat domain 67	8q24.13	124233227–124233523	0,826	8,8E-05	Gained	0,080319015	NA
*GSDMD1*	Gasdermin D	8q24.3	144710202–144710602	-0,821	1,3E-04	Gained	0,107927089	NA
*GPATCH2*	G patch domain containing 2	1q41	215848737–215849264	0,812	1,8E-04	Unspecified	0,137376561	NA
*WSB2*	WD repeat and SOCS box-containing 2	12q24.23	116955326–116955682	0,809	2,1E-04	Unspecified	0,142275217	[42]
*CACNA1C*	Calcium channel, voltage-dependent, L type, alpha 1C subunit	12p13.3	2670999–2671234	-0,803	2,7E-04	Deleted	0,160672631	[40,41]
*KRT79*	Keratin 79	12q13.13	51501640–51502010	-0,800	3,0E-04	Unspecified	0,160672631	NA
*PPHLN1*	Periphilin 1	12q12	41121456–41121979	0,797	3,2E-04	Gained	0,160672631	[43,44]
*C1orf107*	Chromosome 1 open reading frame 107	1q32.2	208096981–208097464	0,794	3,5E-04	Gained	0,160672631	NA
*FBXL14*	F-box and leucine-rich repeat protein 14	12p13.33	1570852–1571348	0,794	3,5E-04	Unspecified	0,160672631	NA
*ANKRD11*	Ankyrin repeat domain 11	16q24.3	87999533–87999771	0,794	3,5E-04	Deleted	0,160672631	[45,52]
*HEATR1*	HEAT repeat containing 1	1q43	234815741–234816376	0,791	3,9E-04	Gained	0,160672631	NA
*COG2*	Component of oligomeric golgi complex 2	1q42.2	228895774–228896323	0,791	3,9E-04	Gained	0,160672631	[39]

^1^Location of the Affymetrix target sequence using BLAST algorithm. ^2^Copy number status = characterization of the copy number status in the whole patient set; unspecified indicates that both amplifications and deletions were found. NA = not available.

### Microarray analysis and quantitative RT-PCR on HDGF

**Figure 4:**
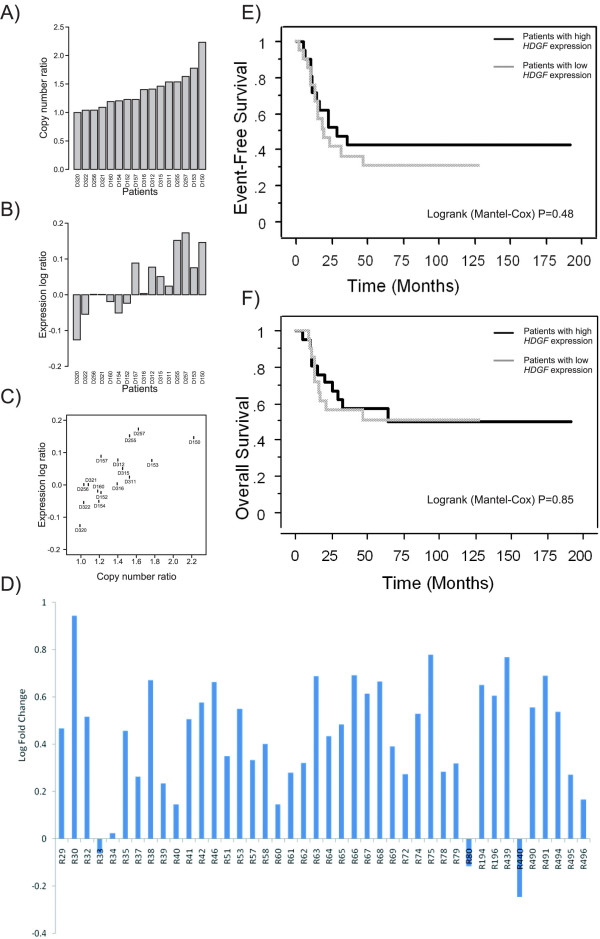
**Correlation of *HDGF *copy number and expression by microarray analysis and validation of *HDGF *expression using RT-PCR**. A) *HDGF *copy number ratio and B) *HDGF *expression ratio by microarray analysis in ESFT patient samples (n = 16). The patients in figure A) and B) are in the same order, and labelled according to the codes of the DNA samples. C) Correlation of *HDGF *copy number and expression ratio (correlation 0.844, P < 0.001, q-value 0.024). D) *HDGF *expression in ESFT patient samples (n = 42) by RT-PCR analysis, on y-axis refers to log of fold-change in *HDGF *gene expression and x-axis to patients RNA code number. Kaplan-Meier plots of ESFT patient (n = 42) survival according to D) event-free survival and E) overall survival, patients with high expression of *HDGF *in bold line and patients with low expression in hatched line.

Array CGH and expression microarray results on showed clear evidence that patients with *HDGF *gain had higher *HDGF *expression (Figure 4A–C, correlation 0.81) than patients without *HDGF *gain. However, ESFT patients could not be divided unambiguously into two groups (see Figure 4A and 4B) based on this data. To validate *HDGF *microarray results, the relative expression levels of *HDGF *were analysed by TaqMan Low Density arrays in all 42 available ESFT patient samples (Figure 4F). This analysis confirmed that ESFT patient samples express higher levels of *HDGF *than normal controls. No statistically significant correlation of *HDGF *expression with poor clinical outcome could be shown (Figure 4D and 4E), nor correlation with patient gender, age or location could be shown. Clinical data summary of these 42 ESFT patients included in the analysis can be viewed on Additional file 2.

## Discussion

In this study, we have performed a comprehensive genome wide array CGH analysis of 31 EFST patient samples. Our oligoarray CGH results, the recurrent gains of 1q, 2, 8, and 12, and losses at 9p and 16q that were present in more than 20% of the patient samples, are in agreement with previous ESFT studies by G-banding, conventional CGH [4-8] and array CGH [9]. Our array CGH results revealed complex large-scale changes in several samples. Gains of DNA sequences were more prevalent than losses and most of the gains affected whole chromosomes or chromosome arms. Further, our analysis showed that patients with low copy number changes (≤ 3 copy number aberrations) showed a significantly better prognosis than patients with a high number of chromosomal alterations, both in terms of event-free and overall survival.

Concomitant gains of 11q24.3-qter and 22q11.21-q12.1 detected in three samples (D153, D248, and D254) suggest that a reciprocal translocation took place between the *EWSR1 *and *FLI1 *loci and thereafter a duplication event of the derivative chromosome 22. Sample D153 had Type 3 *EWS-FLI1 *translocation and sample D248 Type 1 translocation, which suggests that the possible duplication event of the derivative chromosome is not translocation type-specific. In addition, copy number imbalances affecting the *EWSR1 *and/or *FLI1 *loci were detectedin three other samples (D154, D312, and D315). In sample D315, with Type 1 *EWS-FLI1 *translocation, loss of 22q12.1 was observed to end at the *EWSR1 *loci. Similar evidence has been reported previously [12,13,24]. Our results suggest that duplication of the der(22)t(11;22) is a common event in ESFT. Copy number gain of the fusion gene *EWS-FLI1 *may further increase the expression of this fusion product and possibly impair the prognosis. Amplification or gain of a chimeric fusion gene is relatively infrequent mechanism in both leukemia and solid tumors. However, rare cases of gain or amplification of the derivative chromosomes or episomes carrying the fusion gene have been reported [25-27] and gene dosage effect of the fusion gene can improve the tumor growth resulting in more aggressive course of disease [27]. Unfortunately our sample set was not large enough in statistical power to study this aspect.

Interestingly, sample D154, which was negative for *EWS-FLI1 *translocation types that we tested, showed cryptic amplifications on chromosomes 20 and 22 (Figs. 3E and 3F). Szuhai et al. have reported a similar case with 20q and 22q amplifications, suggesting that the translocation partner of *EWSR1 *is at 20q [28]. However, the specific chromosomal region in 20q remained unknown. Based on our results, the translocation partner of *EWSR1 *on chromosome 20 might reside in the amplification breakpoint, either at 20q11.23 or 20q13.12-q13.2 (Fig. 2E). Putative translocation partners of *EWSR1 *are therefore genes assigned to the breakpoints of these amplifications:*RPN2, BLCAP, CDH22, SLC13A3, EYA2, NCOA3, Kua-UEV*, and *NFATC2*. Based on literature, the most interesting candidates are *EYA2 *(located at 20q13.12), which has been found to function as a transcriptional activator in ovarian cancer cells [29], and *NFATC2 *(located at 20q13.2), which functions in positive regulation of transcription [30]. Both *EYA2 *and *NFATC2 *are oriented on the amplification breakpoints so that they are in the correct direction for transcription after the possible fusion event. In addition to the chromosome 20, genes on region 8p are interesting as putative fusion partners, since many of these genes are involved in carcinomas and sarcomas. Indeed the region of 8p11.21-p21.2 was gained in patent sample D154. However, the possible involvement of 8p11.21-p21.2 as a location of the translocation partner for *EWSR1 *was ruled out since this region was not amplified like *EWSR1 *was. We would assume that the fusion partners would be amplified on the same scale, since translocation is likely to take place before the amplification of the fusion gene.

According to our integrated analysis of array CGH and expression data including 16 ESFT patient samples, we selected as one of the most interesting putative target genes within the common 1q22-qter gain gene *HDGF*, which has been reported as a putative prognostic marker for several tumor types, e.g., gastrointestinal stromal tumors (GIST) [31,32], hepatocellular carcinoma [33], non-small-cell lung carcinoma [34,35] and pancreatic ductal carcinoma [36]. *HDGF *has been shown to stimulate cell proliferation and growth after nuclear translocation [37,38], which makes it a likely target also in ESFT. Furthermore, our preliminary results from an aCGH analysis of ESFT cell lines showed that *HDGF *was inside the minimal common overlapping area of 1q21.1-q23.1 (Savola et al, unpublished results). Our RT-PCR analysis confirmed that Ewing's sarcoma cells expressed higher levels of *HDGF *with respect to putative normal controls (CD34 positive cells and normal muscle tissues). However, when we analyzed *HDGF *expression level correlation with patient survival, no significant association was seen. So *HDGF *can play a role in the tumorigenesis and tumor progression of EFST, but it shows no prognostic value. However, due to limitations in numbers of patients (n = 42) included in the *HDGF *expression study, no definitive conclusions of the outcome evaluation of *HDGF *expression in ESFT can be drawn.

Other interesting target genes pinpointed by integration analysis in 1q include *TMEM63A *(1q42.12), *C1orf107 *(1q32.2), *HEATR1 *(1q43), all relatively unknown genes in their functions and *COG2 *(1q42.2), gene involved in various Golgi functions [39]. In chromosome 8 genes *WDR67 *(8q24.13) and *GSDMDC1 *(8q24.3) locating nearby each other and in chromosome 12 *DDX47 *(12p13.1) and *CACNA1C *(12p13.3) [40,41] are interesting targets for further studies. Also potential oncogenes in ESFT at 12q are *WSB2 *(12q24.23), which takes part in the intracellular signalling cascades and has shown to be a potential biomarker in colorectal cancer [42], *PPHLN1 *(12q12), which controls cell cycle regulation by modifying expression of cdc7 involved in progression of DNA replication [43,44] and *KRT79 *(12q13.13), a member of human type II keratin gene family. Previously loss of 16q has been shown to be a sign of poor prognosis in ESFT [7,13]. Our results suggest that the putative target gene within this chromosomal area is *HEATR3 *(16q12.1) or *ANKRD11 *(16q24.3), which has been recently identified to interact with p53 and act as a co-activator in the regulatory feedback loop with p53 [45]. Functional studies to confirm these results are warranted.

## Conclusion

This study adds new information regarding gene copy number changes and their relation to expression in ESFT providing valuable data for further analysis. In addition, array CGH showed to be efficient in the detection of a putative novel translocation in one patient sample and provided new information about copy number changes of the *EWS/FLI1 *fusion gene. Therefore we can conclude that array CGH analysis and integrated DNA microarray analysis of global gene expression patterns and gene copy number imbalances is a powerful method to identify novel molecular targets and chromosomal regions of highest interest in ESFT.

## Competing interests

The authors declare that they have no competing interests.

## Authors' contributions

SS designed the study plan, carried out array analysis, interpreted the data and wrote the manuscript. AK and AT performed bioinformatic analysis in the supervision and coordination of SKaski. TN took part in the array CGH analysis. MS, PP, DZ and KS provided clinical specimens, collected the clinical data, performed and organized *HDGF *analysis by RT-PCR and contributed to the design of study plan. SKnuutila was the principal investigator managing the conception of the study and data interpretation. All authors contributed to the manuscript and approved the final version of it.

## Pre-publication history

The pre-publication history for this paper can be accessed here:

http://www.biomedcentral.com/1471-2407/9/17/prepub

## Supplementary Material

Additional file 1 **Results of integration analysis on RNA and DNA data in ESFT.** Complete results of bioinformatic analysis (gene location, correlation, p-value, q-value and copy number status) on DNA and RNA data integration. Click here for file

Additional file 2 **Clinical data summary of 42 ESFT patients in *HDGF *expression and survival analysis.** Table of clinical characteristics (sex, age, location of tumor, event-free and over all survival) of ESFT patients studied in RT-PCR analysis of *HDGF*. Click here for file
