# Supplementary material for: Combined use of expression and CGH arrays pinpoints novel candidate genes in Ewing sarcoma family of tumors
Source: BMC Cancer. 2009 Jan 14;9:17. doi: 10.1186/1471-2407-9-17 (PMC2633345; doi:10.1186/1471-2407-9-17)
Supplement: Additional file 2 — Clinical data summary of 42 ESFT patients in HDGF expression and survival analysis. Table of clinical characteristics (sex, age, location of tumor, event-free and over all survival) of ESFT patients studied in RT-PCR analysis of HDGF. [file 1471-2407-9-17-S2.doc]

**Additional file 2 - Clinical data summary of 42 ESFT patients in *HDGF* expression analysis. (**REL = relapsed; NED = no evidence of disease)

|  | **Total** |
| --- | --- |
| **n** | 42 |
| **Sex (M/F)** | 25/17 (59.5%/40.4%) |
| **Age** | 27 (>14 years)  15 (<14 years) |
| **Even free survival (EFS)** |  |
| **REL** | 26 |
| **NED** | 16 |
| **Follow-up** | 2 -192.2 months |
| **Mean** | 45.1 months |
| **Median** | 22.5 months |
| **Over all survival (OVS)** |  |
| **Alive** | 22 |
| **Dead** | 20 |
| **Follow-up** | 4.6-192.2 months |
| **Mean** | 55.6 months |
| **Median** | 45.2 months |
| **Location** |  |
| **Extremity** | 26 |
| **Pelvis** | 6 |
| **Other** | 10 |
